# ALDH1A3-regulated long non-coding RNA NRAD1 is a potential novel target for triple-negative breast tumors and cancer stem cells

**DOI:** 10.1038/s41418-019-0362-1

**Published:** 2019-06-13

**Authors:** Dejan Vidovic, Thomas T. Huynh, Prathyusha Konda, Cheryl Dean, Brianne M. Cruickshank, Mohammad Sultan, Krysta M. Coyle, Shashi Gujar, Paola Marcato

**Affiliations:** 1 Department of Pathology, Dalhousie University, Halifax, NS Canada; 2 Department of Microbiology and Immunology, Dalhousie University, Halifax, NS Canada

**Keywords:** Cancer stem cells, Epigenetics

## Abstract

To discover novel therapeutic targets for triple-negative breast cancer (TNBC) and cancer stem cells (CSCs), we screened long non-coding RNAs (lncRNAs) most enriched in TNBCs for high expression in CSCs defined by high Aldefluor activity and associated with worse patient outcomes. This led to the identification of non-coding RNA in the aldehyde dehydrogenase 1 A pathway (NRAD1), also known as LINC00284. Targeting NRAD1 in TNBC tumors using antisense oligonucleotides reduced cell survival, tumor growth, and the number of cells with CSC characteristics. Expression of NRAD1 is regulated by an enzyme that causes Aldefluor activity in CSCs, aldehyde dehydrogenase 1A3 (ALDH1A3) and its product retinoic acid. Cellular fractionation revealed that NRAD1 is primarily nuclear localized, which suggested a potential function in gene regulation. This was confirmed by transcriptome profiling and chromatin isolation by RNA purification, followed by sequencing (ChIRP-seq), which demonstrated that NRAD1 has enriched chromatin interactions among the genes it regulates. Gene Ontology enrichment analysis revealed that NRAD1 regulates expression of genes involved in differentiation and catabolic processes. NRAD1 also contributes to gene expression changes induced by ALDH1A3; thereby, the induction of NRAD1 is a novel mechanism through which ALDH1A3 regulates gene expression. Together, these data identify lncRNA NRAD1 as a downstream effector of ALDH1A3, and a target for TNBCs and CSCs, with functions in cell survival and regulation of gene expression.

## Introduction

Triple-negative breast cancers (TNBCs) represent 15–20% of breast tumors and are associated with worse outcomes [1, 2]. This is in part due to the reliance on chemotherapies to treat these tumors, since they lack hormone receptors and are refractory to hormone receptor antagonists. Transcriptome profiling identifies five major subtypes in breast cancer; luminal A, luminal B, HER2 overexpressing, basal-like, and claudin-low. The majority of TNBCs are basal-like (60–85%). In comparison to other subtypes, TNBC/basal-like breast cancers have higher percentages of cancer stem cells (CSCs) [3–9], which may contribute to the aggressiveness associated with the subtype. CSCs are the most tumorigenic cells in tumors, have stem-like qualities and are commonly defined by increased aldehyde dehydrogenase (ALDH) activity [10]. Most concerning in terms of mitigating the risk of recurrence, is the resistance of CSCs to chemotherapies, radiotherapy, and possibly immunotherapies [11–14]. Given the high abundance of CSCs within TNBC/basal-like breast cancer [3–9], novel therapies that also target CSCs may better reduce the risk of relapse and improve patient outcomes.

CSC-associated enzymes (e.g., ALDHs) and signaling pathways (e.g., Notch, Wnt, and Hedgehog) are also mediators of tumorigenicity, metastasis, and therapy resistance, and may provide avenues for therapeutic intervention [13]. In addition to these protein-coding gene targets, it may also be possible to inhibit CSCs via targeting non-protein-coding gene products. Increasing evidence is demonstrating the function of long non-coding RNA (lncRNAs) in cancer development [15], metastasis [16], and drug resistance [17]. LncRNAs are defined as non-protein-coding transcripts greater than 200 nucleotides. Over 20,000 lncRNAs have been identified in the human genome, but the functions of only hundreds are known, providing a large pool of potential novel therapeutic targets for discovery. In terms of function, characterized lncRNAs act as enhancers of transcription, decoys for transcription factors, guides and recruiters of chromatin-modifying complexes and transcription factors, scaffolds for molecular interactions, or competitive endogenous RNAs (ceRNAs) that bind and sequester (‘sponge’) miRNAs [18]. They are also attractive therapeutic targets because they exhibit polarized tissue-specific expression patterns and tend to be selectively expressed in certain cancers.

The preclinical evidence regarding lncRNA antagonists for the treatment of cancer is promising. Pharmacological inhibition of cancer-specific lncRNAs in vivo (with modified antisense oligonucleotides termed GapmeRs [19]) inhibited tumor growth and metastasis, and sensitized tumors to other therapies [16, 17]. In terms of CSC-specific lncRNAs, only a handful have been found to be more abundant in putative CSC populations and increase stemness features [20]. For TNBC, recent analysis of patient tumor RNAseq data, available from The Cancer Genome Atlas (TGGA), revealed over 50 lncRNAs that are highly enriched in TNBCs/basal-like breast cancers [21]. Among these TNBC/basal-like enriched lncRNAs, LINP1 was identified as a regulator of DNA repair [21]. Aside from LINP1, most of the TNBC/basal-like enriched lncRNAs remain uncharacterized, and some could be functional and serve as novel TNBC targets. Importantly, accumulating evidence is illustrating that pharmacological inhibition of a CSC/TNBC-specific lncRNA may be an effective therapeutic strategy, especially considering recent FDA approval of antisense oligonucleotide-based therapies for the treatment of neurodegenerative disorders [22].

With the goal of identifying a novel oncogenic lncRNA that could be targeted with antisense oligonucleotides to treat TNBCs and kill CSCs within these tumors, we screened for lncRNAs that are enriched in TNBCs and CSCs and are associated with poor patient outcomes. This led to the identification of a previously uncharacterized lncRNA, LINC00284, which hence forth shall be referred to as non-coding RNA in the aldehyde dehydrogenase 1 A pathway (NRAD1). Targeting NRAD1 with antisense oligonucleotides decreased cell viability and reduced tumor growth of TNBC cells lines in a patient-derived xenograft (PDX). Ex vivo analysis of the residual PDX tumors post-treatment revealed fewer live cancer cells with reduced mammosphere formation potential. These results are consistent with gene expression analyses, where NRAD1 upregulates genes involved in catabolism and survival, and downregulates genes involved in differentiation. Functional analyses revealed that NRAD1 is nuclear localized with genome-wide chromatin interactions enriched among the genes it regulates. Finally, NRAD1 is a novel downstream target of aldehyde dehydrogenase 1A3 (ALDH1A3) and the first lncRNA described to contribute to gene expression changes induced by this CSC marker and mediator of tumor progression [23–31]. Together, these data identify the lncRNA NRAD1 as a novel oncogenic effector that is targetable with antisense oligonucleotides in the treatment of TNBC and reduction of cells with CSC characteristics.

## Methods and materials

### Cell lines, cell culture, and the patient-derived xenograft

All cell lines were obtained from the American Type Culture Collection (ATCC), with the exception of SUM149 cells that were obtained from BioIVT (previously Asterand), were cultured as per the supplier’s recommendations. The TNBC patient-derived xenograft (PDX) 7482 was obtained as a low-passage cryopreserved tumor piece from Dr. Michael Lewis (Baylor College of Medicine). Prior to experimentation, the cryopreserved PDX tumor pieces were revived and surgically implanted in the mammary fat pad of a NOD/SCID female mouse for expansion for 5 weeks. PDX 7482 originated from a grade 3, stage 2 primary tumor, breast carcinoma [32]. For retinoic acid treatment, cell lines were treated with 100 nM all-trans retinoic acid (ATRA; Sigma–Aldrich) for 24 h, and then collected for quantitative polymerase chain reaction (QPCR) analysis as described below.

### Quantitative PCR

For all QPCR analyses, cells were collected in TRIzol and RNA purified using a PureLink RNA kit (Invitrogen Thermo Fisher Scientific) following the manufacturer’s instructions. RNA was reverse transcribed with the iScript cDNA Synthesis Kit (Bio-Rad) as per manufacturer’s instructions. QPCR was performed using SsoAdvanced Universal SYBR Supermix (Bio-Rad) and gene-specific primers (primer sequences are listed in Supplemental Table 1) as per manufacturer’s recommended protocol using a CFX96 Touch RealTime PCR Detection System (Bio-Rad). Standard curves were generated for each primer pair, and primer efficiencies were incorporated into the CFX Manager software (Bio-Rad). Gene expression of all samples was calculated relative to reference genes (GAPDH, B2M, PUM1, ARF1, or RPL13A).

### ALDH1A3 knockdown and overexpression

All stably transfected cell lines were cultured with 0.25 µg/mL puromycin (Sigma–Aldrich). SUM149 and MDA-MB-468 ALDH1A3 knockdown clones were generated previously using retroviral pSMP-shRNA vectors (shRNA1, shRNA2) [27]. ALDH1A3-overexpressing MCF7 cells were generated using pMSCVpuro-ALDH1A3 retroviral vector as previously described [27]. Knockdown or overexpression of ALDH1A3 was confirmed by QPCR and western blotting (anti-ALDH1A3, Origene, clone 4E8).

### Knockdown of NRAD1 with GapmeRs and cell viability, apoptosis, and mammosphere formation assays

Transient in vitro knockdown of NRAD1 in MDA-MB-468, SUM149, or MCF7 cells was achieved using 15 nM screening-grade modified antisense oligonucleotide GapmeRs, (Qiagen, formerly Exiqon, sequences listed in Supplemental Table 1) admixed with TransIT-BrCa (MS Biolynx) as per the manufacturer’s instructions. Knockdown of NRAD1was confirmed via QPCR, at 48 h post transfection. Knockdown of NRAD1 was maintained in culture by repeated treatments every 48 h. Effects on cell viability and apoptosis were measured by cell counting via trypan blue exclusion or flow cytometry analysis of annexin-V conjugated to Alexa-Fluor 488 (Invitrogen Thermo Fisher Scientific) and **7**-aminoactinomycin D (7AAD, Biolegend) using a FACSCalibur (BD BioSciences) and FCSExpress 4 RE analysis software (De Novo Software).

Alternatively, to assess effects of knockdown on mammosphere formation potential, 4 × 10^3^ SUM149 cells or 5 × 10^3^ cells from PDX 7482 (obtained from a tumor piece which had been expanded in a NOD/SCID mouse, harvested, collagenase treated, strained, red blood cell lysed, washed, and counted) were seeded in complete MammoCult media (Stemcell Technologies) in technical triplicate replicates in 24-well ultra-low-adherence plates (Corning) [33]. Two hours post-seeding, cells were treated with 15 nM GapmeRs, as described above, to transiently knockdown NRAD1. All resulting spheres greater than 50 μm [34, 35] (defined using the integrated software of a Motic (AE31E) microscope), were manually counted one week later (SUM149 cells) or 3 weeks later (PDX 7482 cells). Additionally, after the in vivo PDX 7482 GapmeRs treatment assay described below (Animal Studies subsection), the residual PDX 7482 tumors were harvested from the mice and were processed as described above to generate single-cell suspensions. Equal numbers of live 5 × 10^3^ PDX 7482 dissociated cells from each of the GapmeR-treated tumors were seeded in the low-adherence plates in complete MammoCult media and were not treated with GapmeRs.

### Aldefluor sorting

Cell analysis and isolation of distinct cell populations using a FACSAria (BD Pharmingen) based on Aldefluor activity (Aldefluor assay kit, StemCell Technologies) was performed as per the manufacturer’s instructions and as previously described [36, 37]. To remove dead cells from the sorted populations, the cells were stained with 7-AAD. Additionally, for sorting the dissociated cells of PDX 7482 tumors, the cells were also stained with allophycocyanin (APC) conjugated anti-H2Kd antibody (Biolegend) to remove mouse cells. Diethylaminobenzaldehyde (DEAB) was added to a sample to verify that an Aldefluor^high^ population of cells had been identified. The resulting sorted Aldefluor^low^ and Aldefluor^high^ cell populations were used in the tumor growth assays described below or for RNA extraction and QPCR analysis as described above.

### Animal studies

All animal studies detailed in this manuscript have been conducted in accordance with the Declaration of Helsinki and the Canadian Council on Animal Care (CCAC) standards. For tumor growth assays of FACS-isolated Aldefluor^high^ and Aldefluor^low^ cells, 5000 or 50,000 cells admixed 1:1 with Matrigel-HC were injected into the fourth inguinal mammary fat pads (left: Aldefluor^high^, right: Aldefluor^low^) of 8-week-old NOD/SCID female mice. Tumor volume was measured with calipers (length∗width∗height/2).

For the NRAD1-targeting studies, in vivo-ready GapmeR#4 or control GapmeR (Exiqon, now under Qiagen) were used. Eight-week-old NOD/SCID female mice were injected with 2 × 10^6^ MDA-MB-468 or SUM149 cells admixed 1:1 with Matrigel-HC (BD BioScience) into the right fourth inguinal mammary fat pad. Once palpable tumors formed, the mice were treated subcutaneously (as per Exiqon in vivo guidelines to maximize distribution in mouse tissues) with 15 mg kg^−1^ control GapmeR or GapmeR#4 twice per week.

For PDX 7482, a low-passage (passage 5) cryopreserved tumor piece (~2 mm^3^) was revived from liquid nitrogen storage and surgically implanted into the mammary fat pad of a female NOD/SCID mouse for expansion. Five weeks later, the expanded tumor (passage 6) was aseptically removed from the euthanized mouse and divided into equally sized tumor pieces (~2 mm^3^) and surgically implanted into the second thoracic mammary fat pad of 8-week-old NOD/SCID female mice. The tumors became palpable two weeks post-surgical implantation and the mice were randomized into treatment groups. Treatment with GapmeRs commenced on day 14 post-surgical implantation of PDX 7482 (passage 6).

### Patient data analyses

LncRNA expression and associated patient survival data were extracted from multiple sources (KMPlotter breast cancer database [38], cBioportal (TCGA Cell 2015) [39–41], and TANRIC online software [42]). RNA-seq data of ALDH1A3 in the TCGA Cell 2015 database was retrieved using cBioportal online software [39–41]. RNA-seq data of NRAD1 (LINC00284), ALDH1A3, or LINC00162 (PICSAR) in the CCLE database was retrieved using the CCLE Broad Institute portal (portals.broadinstitute.org/ccle). GO term analysis was performed using Gene Set Enrichment Analysis software (GSEA) [43].

### Microarray analyses

For microarray analyses, MDA-MB-468 cells were treated with either control GapmeR or GapmeR#4 for 48 h and total RNA purified as described above, and shipped to The Centre for Applied Genomics (TCAG, The Hospital for Sick Kids, Toronto, Canada) for Affymetrix Human Gene 2.0 ST microarray platform analysis. The data were processed with the Transcriptome Analysis Console (Affymetrix) to reveal differential gene expression (GSE118710). In the case of control shRNA vs shRNA1 ALDH1A3 MDA-MB-468 cells, we utilized our previously generated Affymetrix Human Gene 2.0 ST microarray data (GSE103427) [26].

### Chromatin Isolation by RNA purification sequencing

ChIRP-seq (chromatin isolation by RNA purification sequencing) experiments were performed as previously described by Chu et al. [44]. Briefly, tiling antisense oligo probes spanning the NRAD1 sequence were generated using the Stellaris FISH Probe Designer (https://www.biosearchtech.com/support/tools/design-software/stellaris-probe-designer) and HPLC-purified probes were purchased from Bio-Synthesis. MDA-MB-468 cells were cross-linked, lysed and sonicated, and incubated with odd or even probesets, and NRAD1-bound chromatin was retrieved. The purified DNA fragments were sequenced by the TCAG using high throughput next generation sequencing (Illumina HiSeq 2000), with read lengths of ~130 bp. Raw reads were uniquely mapped onto the human reference genome (hg38 assembly) using STAR [45]. Peaks were called using MACS 2.0 [46] and shifted bedgraphs were generated. Reads were screened against the blacklist regions (collection of signal artifacts) in the human genome and overlapping reads were removed. The reads were normalized by finding concordance between even and odd probe lane sequences. For this, a consensus track was generated by taking the lower value of the two at each coordinate. Per base coverage was normalized to a total of 150 M mappable reads. The consensus track was assumed as the true coverage for every coordinate, i.e., true coverage = min (even coverage, odd coverage). A SAM file was generated based on this combined lane. Peaks were called from the SAM file using MACS against the corresponding input with a p-value cutoff of 1 × 10^−5^. Peaks were filtered based on the window size of peak (peak summit), peak length, fold enrichment against input lane >2, average coverage >1.5, and pearson correlation >0.3. Sequences of the top 204 true peaks (ranked by fold enrichment) which were among the NRAD1-regulated genes, were extracted and motifs were analyzed using MEME [47].

### Statistical analyses

All statistical analyses were performed in GraphPad Prism 7. In all cases where two samples were compared, a student’s *t*-test was performed. When three or more samples were compared, a one-way ANOVA with Dunnett’s post-test was performed. Significance is listed as follows: * = *p* < 0.05, ** = *p* < 0.01, *** = *p* < 0.001, **** = *p* < 0.0001).

## Results

### NRAD1 is enriched in TNBC/basal-like breast cancers and CSC populations, and is associated with poorer survival in basal-like breast cancers

To generate a shortlist of candidate lncRNAs that could serve as novel functional targets for TNBCs and the CSCs within these tumors, we identified lncRNAs that fulfilled the following prioritization strategy: (1) highly expressed in TNBC/basal-like patient tumors, (2) enriched in breast CSC populations, and (3) associated with worse patient outcomes. For fulfillment of the first criteria, 50 of the most highly enriched lncRNAs in TNBC/basal-like breast cancer patient tumors had already been identified by analysis of the RNAseq data from TCGA breast cancer patient tumor dataset (Supplemental File 1) [21].

**Fig. 1:**
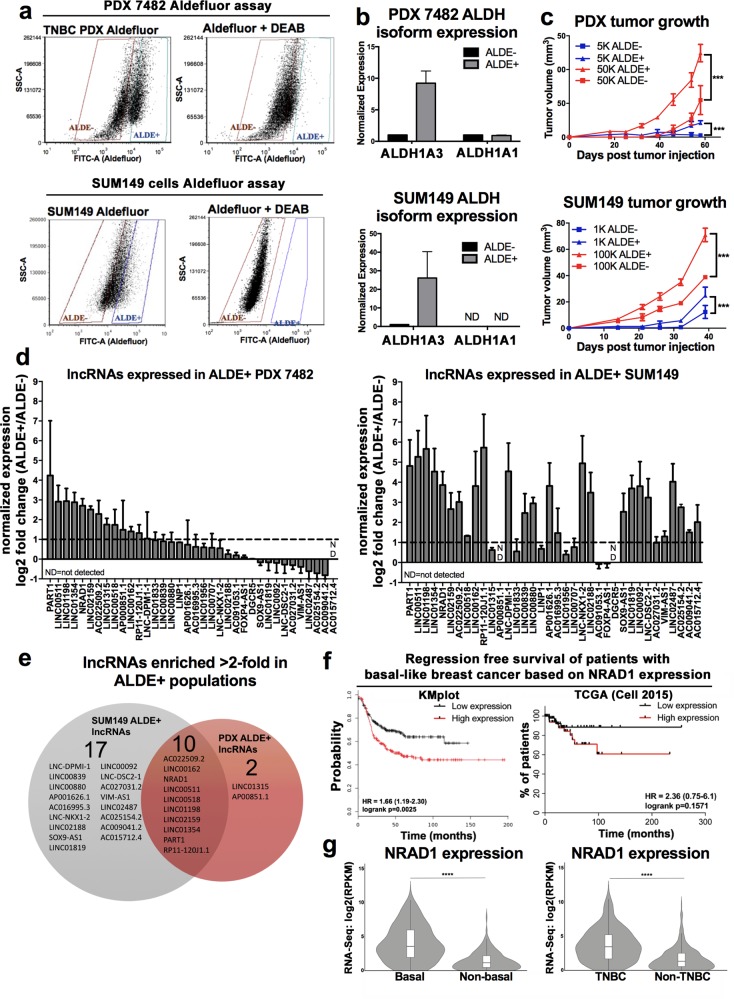
Among TNBC/basal-like enriched-lncRNAs, 10 are enriched in tumorigenic Aldefluor^high^ cells and NRAD1 is also associated with poorer survival in basal-like breast cancers. **a** Aldefluor^high^ (ALDE+) and Aldefluor^low^ (ALDE−) cell populations isolated from PDX 7482 (top) and SUM149 (bottom). The inclusion of a DEAB-treated control confirms that Aldefluor^high^ cells were correctly identified. **b** Transcript levels of ALDH1As in Aldefluor^high^ versus Aldefluor^low^ PDX 7482 or SUM149 cells. **c** Aldefluor^high^ or Aldefluor^low^ sorted populations of TNBC PDX 7482 (top) are assessed for tumor growth potential in mice (*n* = 6 per group for SUM149, *n* = 3 per group for PDX 7482). Error bars represent standard error. **d** Transcript levels of lncRNAs in Aldefluor^high^ versus Aldefluor^low^ PDX7482 cells or SUM149 cells detected by QPCR. The data are normalized to reference genes PUM1 and ARF1 and the log2 fold change over the Aldefluor^low^ mRNA levels (log2 = 0). Error bars represent standard error. **e** A venn diagram shows the number of lncRNAs that were expressed at least 2-fold more (log2 ≥ 1) in Aldefluor^high^ versus Aldefluor^low^ PDX 7482 or SUM149 cells, and commonly in both TNBC models. **f** Regression-free survival in 360 basal-like breast cancer patients based on median expression of NRAD1 was analyzed using KMPlotter [38] (left), and in 106 basal-like breast cancer patients based on median expression of NRAD1 was analyzed using by extracting survival data from TCGA Cell 2015 dataset through cBioportal [39–41] (right). **g** Expression of NRAD1 in TNBC verus non-TNBC patient tumors, or basal-like versus non-basal-like tumors in the TCGA Cell 2015 dataset

Next, we assessed whether any of these TNBC/basal-like enriched lncRNAs were also enriched in the CSCs of TNBCs. For this purpose we utilized two TNBC/basal-like models, PDX 7482 and SUM149 cells, and the Aldefluor assay to identify CSCs among these models [10]. Aldefluor^high^ (ALDE+) and Aldefluor^low^ (ALDE−) cells in the two TNBC models were identified (Fig. 1a). Consistent with the isolation of breast CSCs, the ALDE+ had higher ALDH1A3 levels (Fig. 1b) [36] and greater tumor growth capacity (Fig. 1c) [10]. Having confirmed isolation of cells bearing CSC characteristics, we assessed expression of the 50 TNBC/basal-like enriched lncRNAs (Supplemental File 1) among the sorted tumor samples. Of the 36 lncRNAs we could detect by QPCR (Fig. 1d), 10 lncRNAs were enriched >2-fold (i.e., log_2_ > 1) in the Aldefluor^high^ populations across both TNBC models (Fig. 1e) and were therefore of interest for further analysis.

We compared expression of the 10 TNBC/basal-like/CSC-enriched lncRNAs for correlation with regression-free survival among patients with basal-like breast cancer in multiple breast cancer patient datasets accessed by different portals; KM-plotter [48] (Supplemental Fig. 1, gene array data), cbioportal [40, 41] (Supplemental Fig. 2, TCGA Cell 2015 [39], RNAseq data), and TANRIC [42] (TCGA-BRCA, RNAseq data), and summarized the results of these analyses in Supplemental Table 2. Overall, high expression of NRAD1 (LINC00284) was most consistently associated with decreased survival, although high levels of PART1 and LINC00518 also exhibited some correlations with worse survival (Supplemental Table 2, Fig. 1f). Since genes with oncogenic function are often highly expressed in the tumors of patients with poor outcomes, this is consistent with these lncRNAs possibly having oncogenic function. Among these lncRNAs, NRAD1 best fulfills our criteria for prioritization for functional analysis; a lncRNA that is enriched TNBC/basal-like tumors (Fig. 1g) and CSC populations (Fig. 1a–e) and associated with poor patient outcomes (Fig. 1f).

### NRAD1 confers a survival advantage to breast cancer cells

**Fig. 2:**
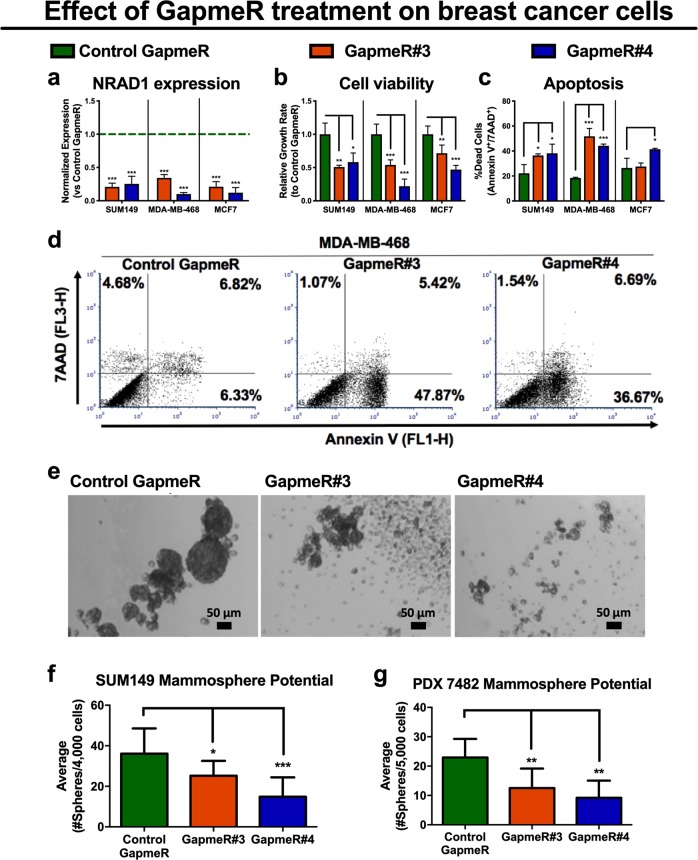
Antisense oligonucelotide targeting of NRAD1 cell viability and the number of cells with mammosphere formation potential. **a** QPCR analysis of NRAD1 expression following knockdown in SUM149, MDA-MB-468, and MCF7 cells treated with NRAD1-specific GapmeRs (#3 or #4) or negative control GapmeR (*n* = 4). Expression is shown normalized to reference genes B2M and GAPDH and relative to the negative control GapmeR-treated sample (set at 1). **b** The effect of NRAD1 knockdown via two different specific GapmeRs versus the control GapmeR was quantified by counting the relative number of viable cells after treatment with GapmeRs, using a trypan blue exclusion assay (*n* = 4). **c** Representative flow cytometry dot plots of MDA-MB-468 cells stained with 7-AAD and annexin V-488 treated with either negative control GapmeR or NRAD1-specific GapmeR#3 or #4. **d** The average percentage of flow cytometry quantified apoptotic SUM149, MDA-MB-468, and MCF7 cells (annexin V and 7-ADD positive cells, *n* = 4). Significance was determined using a one-way ANOVA with Dunnett’s post-test. Error bars represent standard deviation. **e **Representative of images of spheroids that formed after SUM149 cells were seeded in ultra-low nonadherent plates in Mammocult media and treated with either negative control GapmeR or NRAD1-specific GapmeR#3 or #4. Scale bars = 50 μm. **f, g** The average number of resulting spheroids SUM149 cells (**f**, *n* = 6) or PDX 7482 (**g**, *n* = 5) under indicated GapmeR treatment conditions (spheroids greater than 50 μm in size were counted). Significance was determined using one-way ANOVA with Dunnett’s post-test. Error bars represent standard deviation

We first confirmed that NRAD1 is non-coding based on five metrics (Supplemental Table 3). Consistent with the breast cancer patient tumor data (Fig. 1g), NRAD1 is predominately expressed in basal-like breast cancer cell lines (e.g., SUM149 and MDA-MB-468) and is lowly expressed in cell lines of other subtypes (e.g., ER^+^ MCF7 cells, Supplemental Fig. 4), and normal tissues (Supplemental Fig. 5). Although NRAD1 expression was the highest in the TNBC HCC1599 cell line, we did not use this model because the cells grow as aggregates in suspension, making quantification of growth changes difficult, and our attempts to establish tumors in NOD/SCID mice with this cell line failed. Therefore, for functional assessment of NRAD1, we chose two other TNBC basal-like cell lines which form tumors in mice, grow easily in cell culture, and are well-studied (i.e., SUM149 and MDA-MB-468 cells). We also included the ER + MCF7 cells (which have the highest NRAD1 expression among assessed ER+ cell lines) in our functional analysis to serve as an ER+ cell line model, which may reveal if NRAD1 function is limited to TNBC. We transiently knocked down expression of NRAD1 in the cell lines using locked nucleic acid (LNA) GapmeRs, which are antisense oligonucleotides (Fig. 2a). The decrease in NRAD1 levels following knockdown was associated with decreased cell viability (Fig. 2b) and increased apoptosis (Fig. 2c, d). The results with MCF7 cells suggest that growth inhibition from reducing NRAD1 is not exclusive to TN basal-like breast cancers; however, based on the apoptosis results (Fig. 2c), targeting the lncRNA seems less effective in the ER + MCF7 cells with lower levels of NRAD1 expression (Supplemental Fig. 4).

We assessed whether the decrease in general cell survival following NRAD1 knockdown extended to spheroid-forming cells (i.e., an in vitro readout of stemness and tumorigenicity [49, 50]). Transient NRAD1 knockdown achieved with anti-NRAD1-specific GapmeRs decreased spheroid-forming potential of both SUM149 cells (Fig. 2e, f) and in primary cells from PDX 7482 tumors (Fig. 2g). Together, these data suggest that NRAD1 inhibition is detrimental to both cancer cells and stem-like cells with spheroid-forming potential. Furthermore, given the low abundance of NRAD1 in normal tissues (Supplemental Fig. 5), targeting oncogenic NRAD1 for the treatment of TNBC and reduction of CSCs may be a therapeutically viable strategy.

### Therapeutic inhibition of NRAD1 reduces TNBC tumor growth and sphere forming potential of residual tumor cells post-treatment

**Fig. 3:**
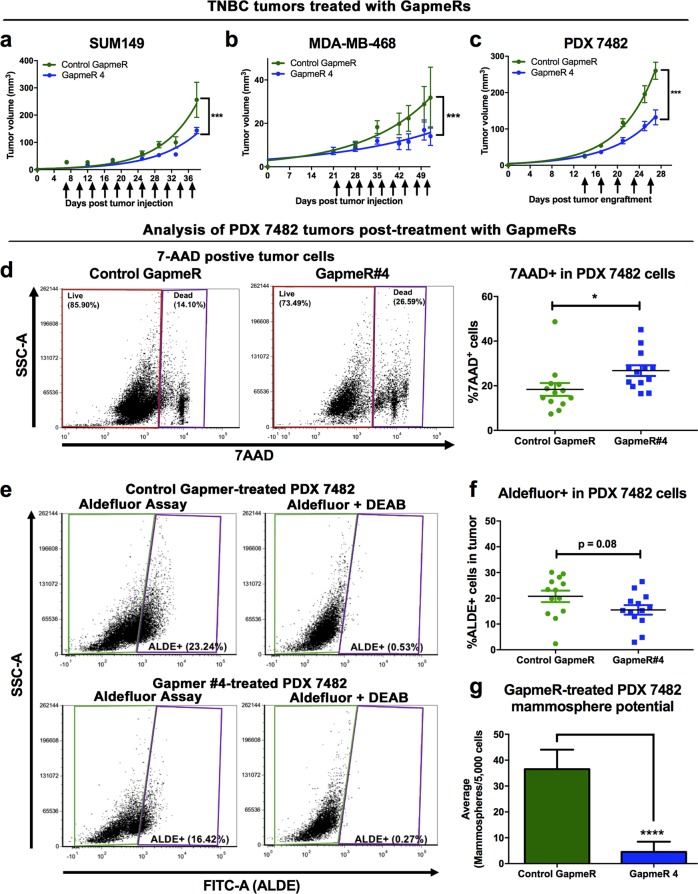
Therapeutic inhibition of NRAD1 reduces breast tumor growth and the remaining tumor cells have limited mammosphere formation potential and reduced Aldefluor activity. **a–c** Resulting tumor volumes of NOD/SCID mice orthotopically implanted with SUM149 cells (**a**, *n* = 6), MDA-MB-468 (**b**, *n* = 6) or 2 mm^3^ PDX 7482 tumor pieces (**c**, *n* = 13) in their mammary fat pads and once palpable tumors developed, treated with either negative control GapmeR or NRAD1-specific GapmeR#4. Arrows indicate when GapmeR treatment occurred. Tumor volume significance was modelled using exponential regression. **d–g** Analysis of post-treatment PDX 7482 tumors. Single-cell suspensions of the harvested tumors were generated and red blood cell lysed, and debris and mouse cells eliminated for FACS analysis. **d** The average number of dead 7-ADD positive cells (representative dot plots, left). **e, f** Of the live the cells (7-AAD negative), the average percentage of Aldefluor^high^ cells was determined (**e**, representative dot plots). **g** An equal number of live cells seeded for mammopshere formation. Significance was determined using student’s *t*-test. Error bars represent standard error

To assess the therapeutic potential of targeting NRAD1, we treated NOD/SCID mice bearing palpable MDA-MB-468, SUM149, or PDX 7482 tumors with control GapmeR or NRAD1-targeting GapmeR #4 after the tumors were established. We chose GapmeR#4 over GapmeR#3 since overall it gave the better NRAD1 knockdown and resulted in more significant effects on growth reduction and apoptosis induction (Fig. 2). Anti-NRAD1 GapmeR treatment significantly reduced the rate of tumor growth in all three TNBC models (Fig. 3a–c). We then assessed the composition of the residual PDX 7482 tumors post-treatment. The smaller anti-NRAD1-treated tumors possessed more dead tumor cells (Fig. 3d), consistent with the observed effect on apoptosis when NRAD1 is silenced in vitro (Fig. 2c, d). The remaining live tumor cells exhibited an insignificant reduction in Aldefluor activity (Fig. 3e, f) and a significant reduction in spheroid-forming potential (Fig. 3g). Together, these data demonstrate that targeting NRAD1 reduced the tumor growth of established TNBC tumors, and the number of cells with CSC-like characteristics within these tumors.

### NRAD1 is a novel downstream target of ALDH1A3 and retinoic acid

**Fig. 4:**
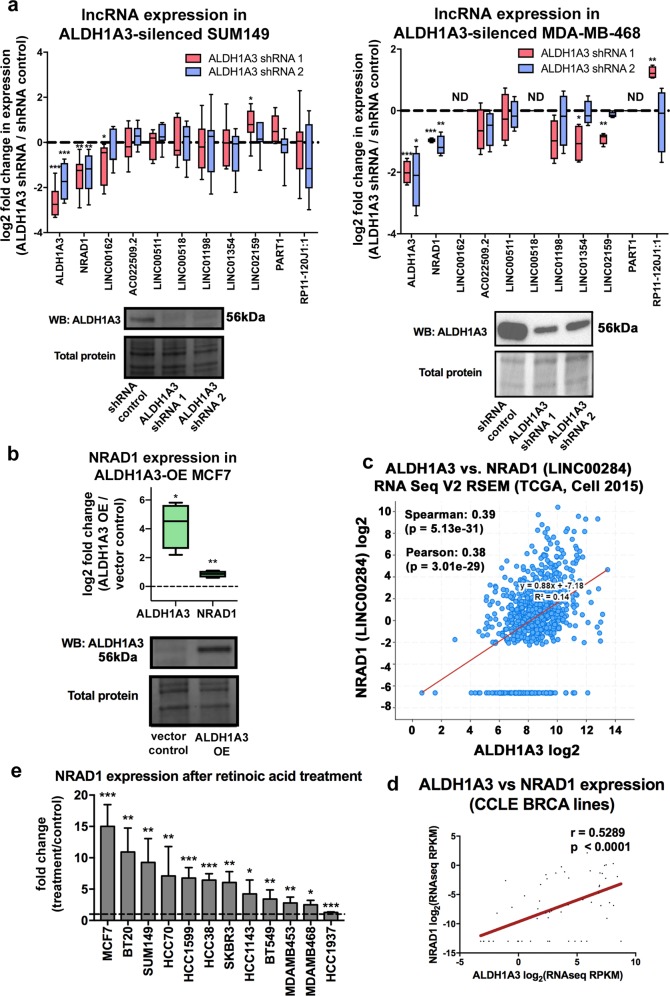
NRAD1 is regulated by ALDH1A3 and all-trans retinoic acid. a QPCR analysis of expression of NRAD1 and the other Aldefluor^high^-enriched lncRNA in SUM149 cells (*n* = 7) or MDA-MB-468 cells (*n* = 4) following knockdown of ALDH1A3 with two separate shRNAs. ND = not detected (levels below quantification threshold). **b** QPCR analysis of ALDH1A3 and NRAD1 levels following overexpression (OE) of ALDH1A3 in MCF7 cells (*n* = 4). **a, b** Transcript levels are shown normalized to reference genes PUM1 and ARF1 and then log2 fold change over control samples. Western blot confirming changes in ALDH1A3 levels is below, using total protein as a loading control (Supplemental Fig. 9 shows uncropped blots). **c** RNA-seq co-expression of NRAD1 and ALDH1A3 in the TCGA Cell 2015 dataset (all breast cancer patients) was retrieved using cBioportal. **d** RNA-seq co-expression of NRAD1 and ALDH1A3 in the Cancer Cell Line Encyclopedia (only breast cancer cell lines) was retrieved using the CCLE portal. **e** QPCR analysis of NRAD1 levels in a panel of cell lines treated with 100 nM retinoic acid for 24 h versus no treatment (*n* = 4). Significance was determined using a one-way ANOVA with Dunnett’s post-test (**a, e**), or a student’s *t*-test (**b**). **c, d**, Spearman and Pearson correlations are shown, and significance was determined using linear regression. All error bars represent standard deviation

Given the co-expression of NRAD1 with CSC marker ALDH1A3 in the Aldefluor^high^ cells (Fig. 1) and the role of ALDH1A3 in gene expression regulation and tumor progression [27], we wondered if NRAD1 is regulated by ALDH1A3. We assessed expression of NRAD1, as well as the other 9 Aldefluor^high^-enriched lncRNAs (Fig. 1e) in SUM149 and MDA-MB-468 cells with or without ALDH1A3 knockdown (Fig. 4a). NRAD1 was unique among the 10 CSC-enriched lncRNAs in that only its expression was significantly downregulated by both ALDH1A3 shRNAs in SUM149 and MDA-MB-468 cells. This suggests that high levels of NRAD1 in Aldefluor^high^ cells may be ALDH1A3-dependent. In a confirmation assay, ALDH1A3 overexpression in MCF7 cells (which have low levels of ALDH1A3) [36], resulted in a corresponding increase in NRAD1 levels (Fig. 4b). Importantly, this association went beyond the three manipulated cell lines, as NRAD1 levels correlated with ALDH1A3 in breast cancer patient tumors (Fig. 4c) and in a panel of breast cancer cell lines (Fig. 4d). In addition to the regulation of NRAD1 by ALDH1A3, our analyses provided some evidence of potential regulation of LINC00162 by ALDH1A3 (i.e., knockdown of ALDH1A3 via shRNA1 reduced expression of LINC00162 in SUM149 cells, Fig. 4a). However, the less efficient ALDH1A3 knockdown (shRNA2) did not affect LINC00162 levels, and we failed to detect LINC00162 in MDA-MB-468 cells with high levels of ALDH1A3 (Fig. 4a). In breast cancer patient tumor samples and in a panel of cell lines, LINC00162 levels did correlate with ALDH1A3 mRNA (Supplemental Fig. 6); however, to a decreased degree than NRAD1 (Fig. 4c, d). Together, these data identify NRAD1 as the first lncRNA actively regulated by a CSC marker (i.e., ALDH1A3), and intimately links NRAD1 with CSCs. To mechanistically investigate this relationship further, we treated a panel of breast cancer cell lines with the ALDH1A3 product all-trans retinoic acid (ATRA), a nuclear receptor ligand and gene expression induction molecule. This uniformly resulted in increased NRAD1 (Fig. 4e). These results demonstrate that NRAD1 is a novel downstream target of CSC marker ALDH1A3 and the RA signaling pathway.

### NRAD1 is nuclear localized and regulates expression of genes in common with ALDH1A3

**Fig. 5:**
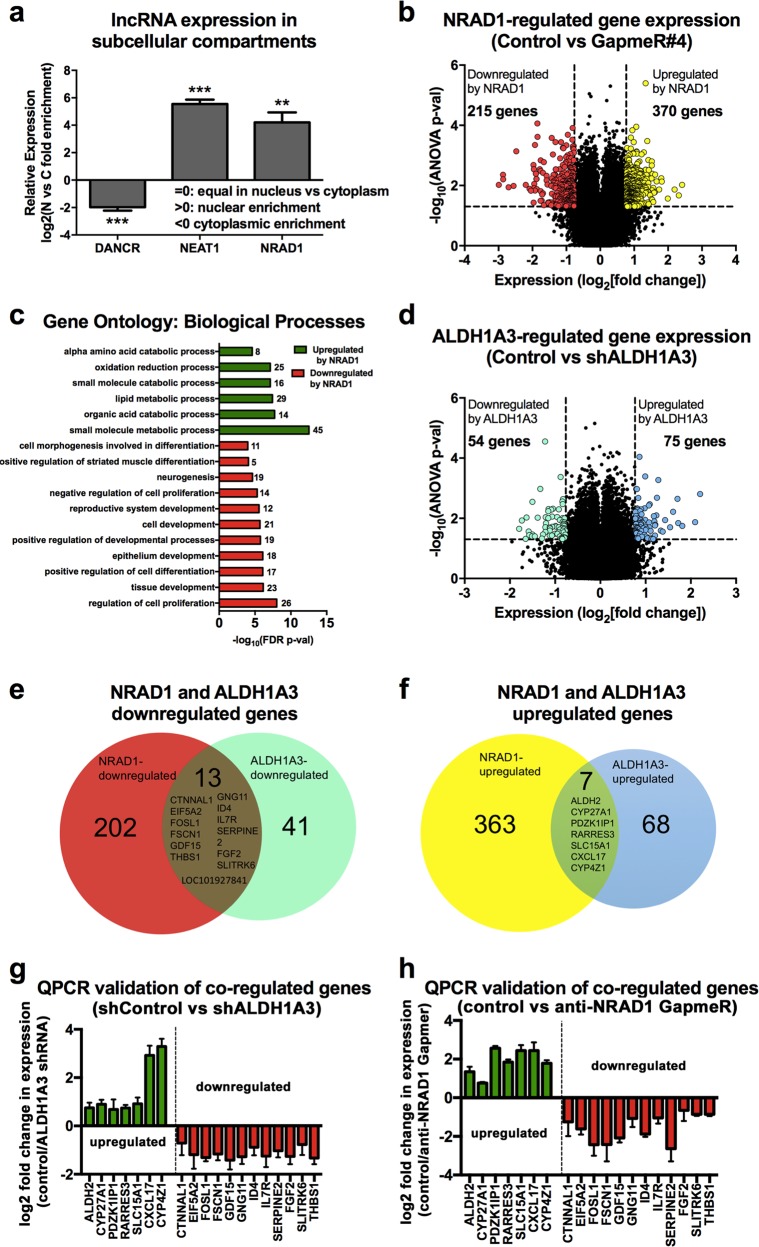
NRAD1 is predominately nuclear and regulates expression of genes, some of which are also regulated by ALDH1A3. **a** QPCR analysis of lncRNA DANCR, NEAT1, and NRAD1 abundance in nuclear and cytoplasmic fractions of MDA-MB-468 cells. Relative expression versus GAPDH is shown (*n* = 3). Significance was determined using student’s t-test, error bars represent standard deviation. **b**, **d** Genome-wide gene expression changes induced by NRAD1 knockdown (**b**, control GapmeR versus GapmeR#4-treated) or ALDH1A3 knockdown (**d**, shRNA control versus ALDH1A3 shRNA1) is quantified in MDA-MB-468 cells using the Affymetrix Human Gene 2.0 ST microarray platform (*n* = 3). The log_2_-fold change in expression is plotted versus the –log_10_(ANOVA p-val) of over 50,000 probes corresponding to 24,838 probesets covering 24,838 RefSeq (Entrez) genes. Only probes with a > 1.70-fold expression change and a p-value of >0.05 are indicated as colored dots. **c** Gene ontology (GO) terms analysis was performed on NRAD1 up- or downregulated genes using GSEA software. The most significant GO terms with high numbers of genes enriched in those pathways are shown. **e**, **f** Venn diagrams showing the number of genes downregulated (**e**) or upregulated (**f**) by NRAD1 and ALDH1A3, with the genes co-regulated by both in the center. **g**, **h** QPCR validation of the NRAD1 and ALDH1A3 co-regulated genes in MDA-MB-468 cells. Log2 fold change of transcript levels in ALDH1A3 shRNA2 versus control shRNA (**g**) or NRAD1-specific GapmeR#4 versus control GapmeR (**h**). Expression is normalized to reference genes PUM1 and ARF1 and represented as fold change over control cells (*n* = 4). Error bars represent standard deviation (n.d. = not detected)

Determining cellular localization is an informative first step for characterizing potential lncRNA functions [51]. Fractionation of MDA-MB-468 cells revealed that NRAD1 is predominately nuclear, like positive control nuclear-localized lncRNA NEAT1 [19], and in contrast to cytoplasmic-localized lncRNA DANCR [19] (Fig. 5a, Supplemental Fig. 7). Given that nuclear lncRNAs often function in gene expression regulation [51], we performed microarray transcriptome analysis of MDA-MB-468 cells with or without NRAD1 knockdown by GapmeR#4. We chose GapmeR#4 for the microarray transcriptome analyses as it was more effective than GapmeR#3 at silencing NRAD1 in MDA-MB-468 cells (Fig. 2a). This revealed 370 genes with decreased expression upon NRAD1 knockdown (i.e. NRAD1-upregulated genes) and 215 genes with increased expression (i.e. NRAD1-downregulated genes) (Fig. 5b, Supplemental File 2). QPCR validation of a representative sampling of the microarray-identified genes confirmed their regulation by NRAD1 using both GapmeR#3 and #4 to decrease levels of NRAD1 in MDA-MB-468, SUM149, and MCF7 cells (Supplemental Fig. 8). The regulation of these selected genes by NRAD1 was more evident in MDA-MB-468 and SUM149 cells (Supplemental Fig. 8A, B) in comparison to MCF7 cells (Supplemental Fig. 8C). This may reflect the less pronounced effect targeting NRAD1 has on the cell growth and apoptosis of MCF7 cells compared to MDA-MB-468 and SUM149 cells (Fig. 2b, c). Gene ontology (GO) term enrichment analysis revealed that NRAD1 downregulates genes involved in developmental and differentiation processes, and upregulates genes involved in alpha amino acid metabolism and lipid metabolism (Fig. 5c, Supplemental File 2). This is consistent with the decreased cell viability and reduced spheroid-potential of cancer cells post knockdown of NRAD1 (Fig. 2).

Given that our analyses revealed that NRAD1 is a downstream target of ALDH1A3, we considered if its gene expression regulation contributes to the gene regulation associated with ALDH1A3 [26]. We analyzed our other MDA-MB-468 transcriptome data in which ALDH1A3 had been knocked down and applied the same cutoffs (Fig. 5d, Supplemental File 2). This revealed that a significant number of NRAD1-regulated genes are ALDH1A3-regulated genes (i.e., 24% of ALDH1A3-downregulated genes and 10% of the upregulated genes, Fig. 5e, f). These genes were validated by QPCR using a second ALDH1A3 shRNA (Fig. 5g) and with anti-NRAD1-GapmeR-treated cells (Fig. 5h). Random probability would predict less than 1% gene overlap between the two datasets. This suggests that ALDH1A3 and NRAD1 effects in gene expression are related, and that induction of NRAD1 is a novel mechanism by which ALDH1A3 mediates gene expression changes.

### Genomic occupancy of NRAD1 is enriched among NRAD1-regulated genes

**Fig. 6:**
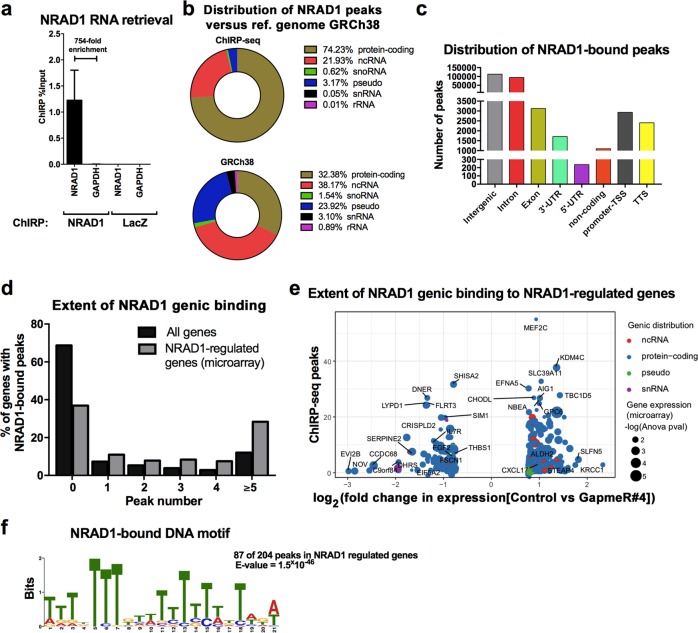
NRAD1-regulated genes have enriched chromatin interactions with the lncRNA. **a** QPCR on the biotinylated probe-bound fraction confirms that the ChIRP assay successfully enriches NRAD1 RNA transcripts over input, versus a GAPDH negative control or LacZ-ChiRP. Error bars represent standard error. **b** In comparison to reference genome hg38, NRAD1 chromatin binding is enriched in protein-coding genes. **c** ChIRP-seq analysis shows the distribution of NRAD1-bound peaks in genic regions. **d** The percentage of genes that have NRAD1 genomic peaks identified by ChiRP-seq in the reference genome (hg38) to the 585 NRAD1-regulated genes identified by microarray. **e** The 369 NRAD1-regulated genes (identified in the microarray, Fig. 5, >1.70-fold expression change and a p-value of >0.05) that have NRAD1 chromatin binding, were plotted according to the log_2_ fold change in expression versus the number of NRAD1-bound peaks. The –log_10_(ANOVA p-value) from the gene expression microarray data indicated by circle size. **f** MEME analysis reveals the motif consensus sequence associated with NRAD1 genomic interactions identified by ChiRP-Seq

Nuclear lncRNAs like NRAD1 (Fig. 5a) often regulate gene expression through chromatin interaction [51]. We performed chromatin isolation by RNA purification (ChIRP) assays in MDA-MB-468 cells to retrieve chromatin bound to NRAD1. We confirmed that only biotinylated probes specific to NRAD1, and not LacZ control, enriched NRAD1 and not non-specific transcript GAPDH (Fig. 6a). Interestingly, analysis of the retrieved DNA fragments by deep sequencing (ChIRP-seq) revealed enriched NRAD1 chromatin interactions among protein-coding genes (Fig. 6b). NRAD1 chromatin interactions were genome wide, and in genic regions, it was particularly abundant among intronic regions (Fig. 6c, Supplemental File 3). Unlike the reference genome in which most genes do not have NRAD1 occupancy, most of the NRAD1-regulated genes (369 of the 585 genes identified in Fig. 5b) had NRAD1 chromatin interactions (Fig. 6d, Supplemental File 3). The enrichment of NRAD1 chromatin binding among the genes it regulates is highly suggestive of its chromatin binding having functional consequences on gene expression. Among the genes regulated by NRAD1, showing the most chromatin binding was transcription factor MEF2C (myocyte transcription enhancer factor 2C, Fig. 6e). It is foreseeable that induction of transcription factors like MEF2C by NRAD1 could lead to the regulation of other genes and may explain the regulation of some of the NRAD1-regulated genes that lack chromatin interactions with the lncRNA (Fig. 6d).

To identify genomic motifs enriched for NRAD1 binding, we assessed the 369 NRAD1-regulated genes with NRAD1 chromatin interactions for conserved motifs using MEME analysis software [47]. We isolated the sequences with a total peak length of 400 bp (+/− 200 base pairs around the peak summit) and sorted by peak fold-enrichment. This revealed 204 peak sequences that were submitted to MEME analysis, revealing a number of highly conserved NRAD1-bound motifs throughout NRAD1-regulated genes. Of note, 87 of the 204 peaks contained a 21 bp T-rich motif (Fig. 6f), which was present in intronic regions. Together, this comparative analysis of the ChIRP-seq and microarray gene expression data suggests that chromatin interactions with NRAD1 leads to direct gene expression regulation by the lncRNA through conserved genic motifs.

## Discussion

Our screening of lncRNAs led to the identification of TNBC/basal-like/CSC-enriched NRAD1 as a new mediator of cell survival within these tumors and cancer cells. Functional characterization revealed that NRAD1 is nuclear localized and binds chromatin leading to changes in gene expression. Targeting the lncRNA with antisense oligonucleotides reduced TNBC tumor growth and the cells within these tumors that have CSC characteristics [20]. NRAD1, therefore, joins a shortlist of lncRNAs that have been described as functionally associated with CSCs. For example, lncRNAs HOTAIR, MALAT-1, linc-ROR, lncRNA-Hh, lincRNA-21, LINC00617, and HULC in the putative CSCs of cell lines increased self-renewal and tumorigenicity [52–59]. The lncRNA TUG1 is upregulated in glioblastoma CSCs and promotes their self-renewal by sponging miR-145 and recruiting polycomb repressive complex 2 (PRC2) to repress genes required for differentiation [60]. NRAD1 is unique among this list in that its expression is regulated by CSC marker ALDH1A3, and hence, it is intimately tied to CSC populations.

ALDH1A3 is the primary contributor of the Aldefluor^high^ activity that defines the CSCs of multiple cancers, is associated with poor prognosis, actively promotes tumor growth, invasion, and metastasis, contributes to chemoresistance in multiple cancers, and has been proposed as a therapeutic target [23, 25, 27–31, 36, 61–70]. ALDH1A3’s tumor-promoting activities are in part mediated by its generation of retinoic acid and subsequent gene expression changes; [27, 71] however, it is unclear which are ALDH1A3’s key downstream oncogenic effectors. Identifying these effectors would delineate the mechanism of ALDH1A3 in cancer. Furthermore, since ALDH1A3 has physiological functions, targeting its key downstream cancer-specific effectors may be better tolerated than targeting ALDH1A3 directly. The data presented here suggest that ALDH1A3/retinoic acid-induced NRAD1 is one of these key oncogenic targetable factors. With recent FDA approval of antisense oligonucleotides for the treatment of neurodegenerative disorders [22], targeting NRAD1 with antisense oligonucleotides for the treatment of TNBC and the reduction of CSCs is a possibility.

Our analyses also revealed that NRAD1 is predominately nuclear and has genomic interactions. Although not all genomic interactions are functional, the enrichment of chromatin interactions among NRAD1-regulated genes suggests that the genomic occupancy of the lncRNA has functional consequence. Among genic regions, the NRAD1 interactions are most common in intronic regions, suggesting the presence of regulatory regions in the introns. Future experiments will reveal if NRAD1 chromatin binding alters chromatin structure and the full importance of NRAD1 in gene regulation, particularly with respect to TNBC/basal-like breast cancers and CSCs where it is most abundant and contributes to cancer cell survival and tumor growth.

## Supplementary information

Supplemental Figures and Tables

Supplemental File 1

Supplemental File 2

Supplemental File 3
