# Supplementary material for: ALDH1A3-regulated long non-coding RNA NRAD1 is a potential novel target for triple-negative breast tumors and cancer stem cells
Source: Cell Death Differ. 2019 Jun 13;27(1):363–78. doi: 10.1038/s41418-019-0362-1 (PMC7206030; doi:10.1038/s41418-019-0362-1)
Supplement: Supplementary file 1 — Supplemental Figures and Tables [file 41418_2019_362_MOESM1_ESM.pdf]

Supplemental Files, Figures and Tables

Supplemental File 1. LncRNAs enriched in basal-like and TNBC patient tumors were assessed for detection in SUM149 cells and PDX 7482 by QPCR. TNBC versus non-TNBC or basal-like vs non-basal breast cancer RNA-seq expression of 48 lncRNAs previously identified as being enriched in TNBC and basal-like breast cancer by Zhang et al., *Nat. Struct. Mol. Biol.* **23,** 522–530 (2016) were assessed for detection by QPCR in SUM149 cells and PDX 7482.

Supplemental File 2. Genes regulated by NRAD1 or ALDH1A3 in MDA-MB-468 cells, and NRAD1-regulated genes identified in Gene Ontology (GO) term enrichment analysis processes.

Supplemental File 3. NRAD1 ChIRP-seq peaks.

Supplemental Figures

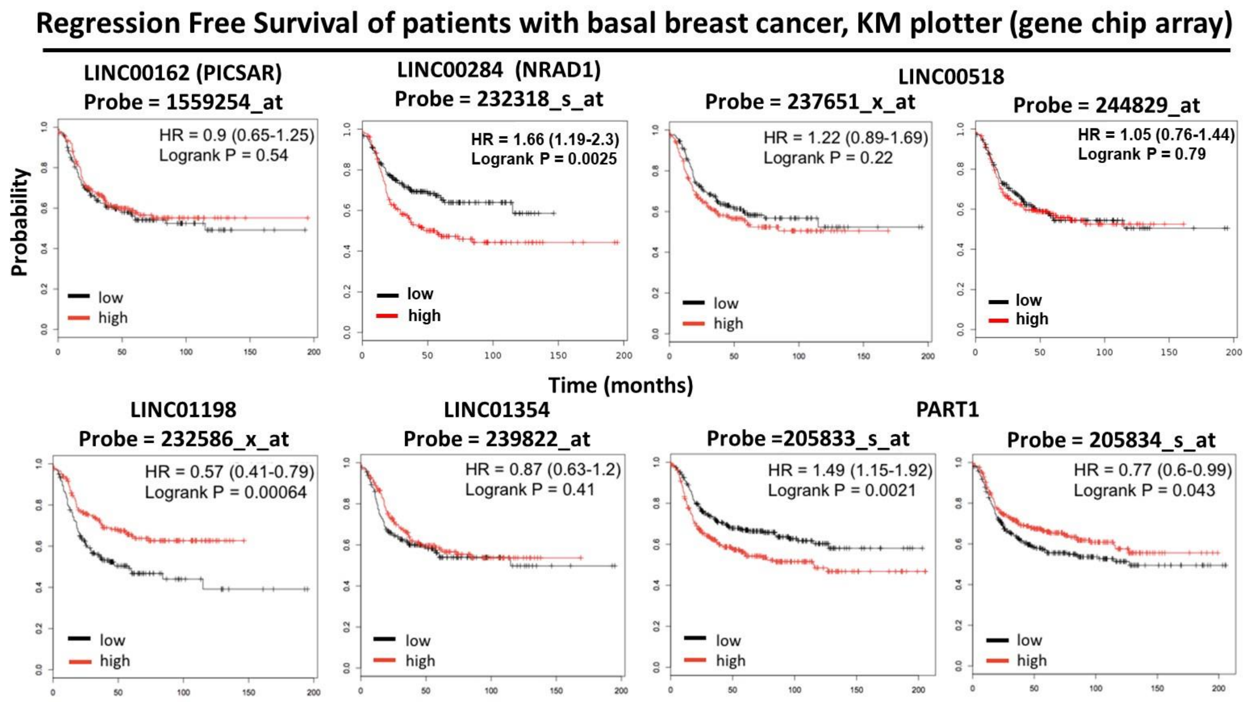

Supplemental Figure 1. KM plotter Regression Free Survival analysis of lncRNAs enriched in CSC populations. Of the 10 lncRNAs identified in Figure 1E, six of the lncRNAs had probes in breast cancer gene chip array data and clinical data compiled by KM plotter. Regression Free Survival based on median expression in 360 basal-like breast cancer patient tumors assessed with KM Plotter (gene chip array). For lncRNAs where more than one probe set is available, the data for all available probe sets is shown. HR = hazard ratio.

Overall survival of patients with basal breast cancer, TCGA Cell 2015, cBioportal (RNA Seq)

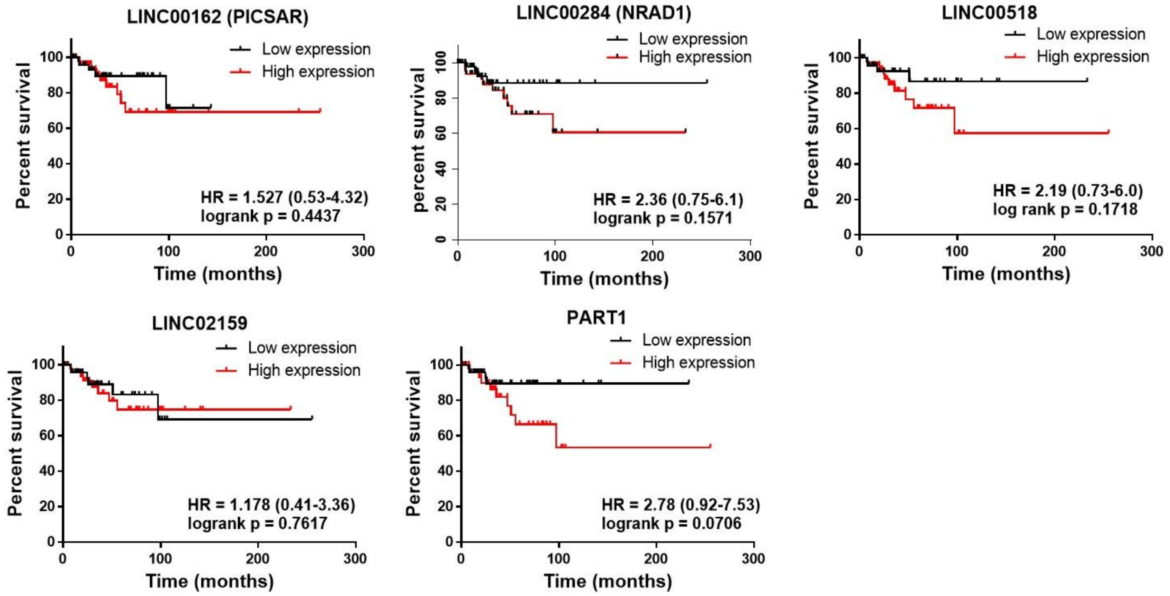

Supplemental Figure 2. TCGA (breast invasive carcinoma, Cell 2015 dataset, cbioportal) overall survival analysis of lncRNAs enriched in CSC populations. Of the 10 lncRNAs identified in Figure 1E, five of the lncRNAs had RNAseq expression data that was extractable by cBioportal from the TCGA, breast cancer Cell 2015 dataset. The overall survival for the 107 patients with basal invasive ductal carcinoma was plotted based on expression of the lncRNAs and their associated clinical data that was also extracted from cBioportal. The patients were divided into high or low expression based on being in the top or bottom half and the survival plots were generated with Graphpad Prism software. HR = hazard ratio (log-rank). The p-value was calculated based on the log-rank (Mantel-Cox) test.

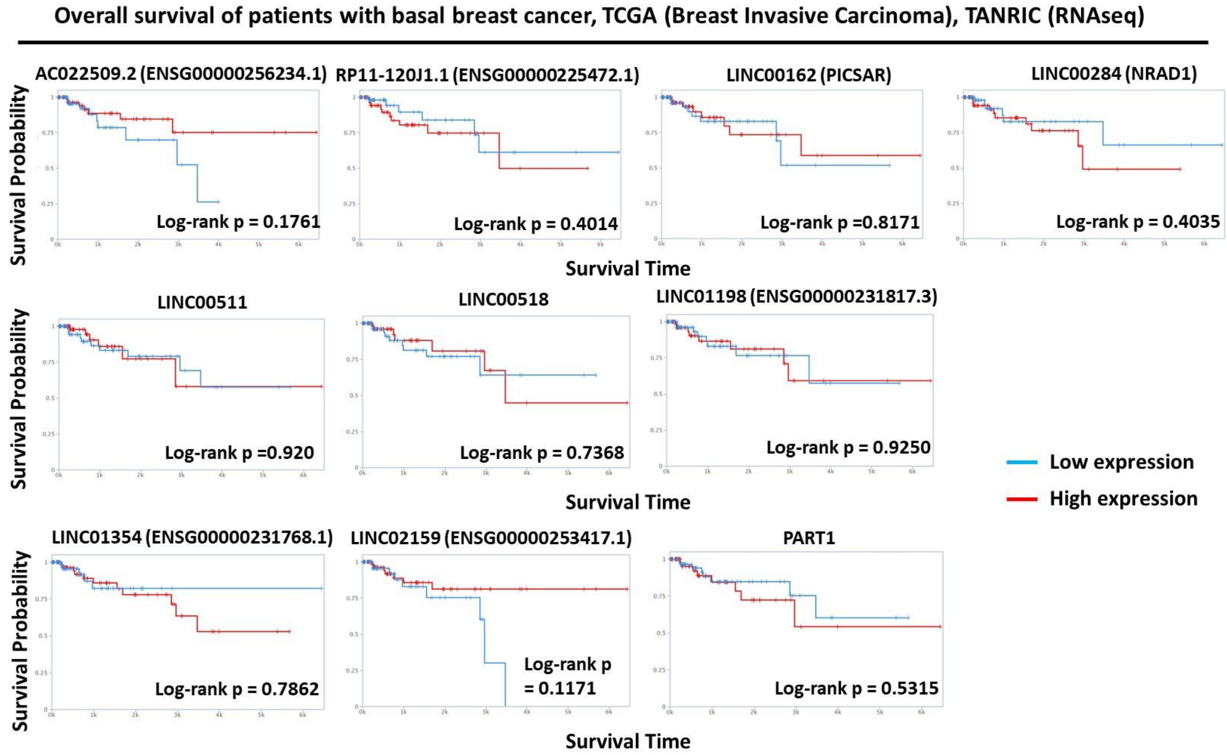

Supplemental Figure 3. TCGA (Breast Invasive Carcinoma, BRCA dataset) overall survival analysis of lncRNAs enriched in CSC populations. Using the TANRIC portal, expression of the 10 lncRNAs identified in Figure 1E were assessed for correlations with overall survival in 139 basal breast cancer patients that are part of the TCGA-BRCA dataset. The survival plots were generated in TANRIC and exported from TANRIC. The hazard ratio was not included in the analysis by TANRIC.

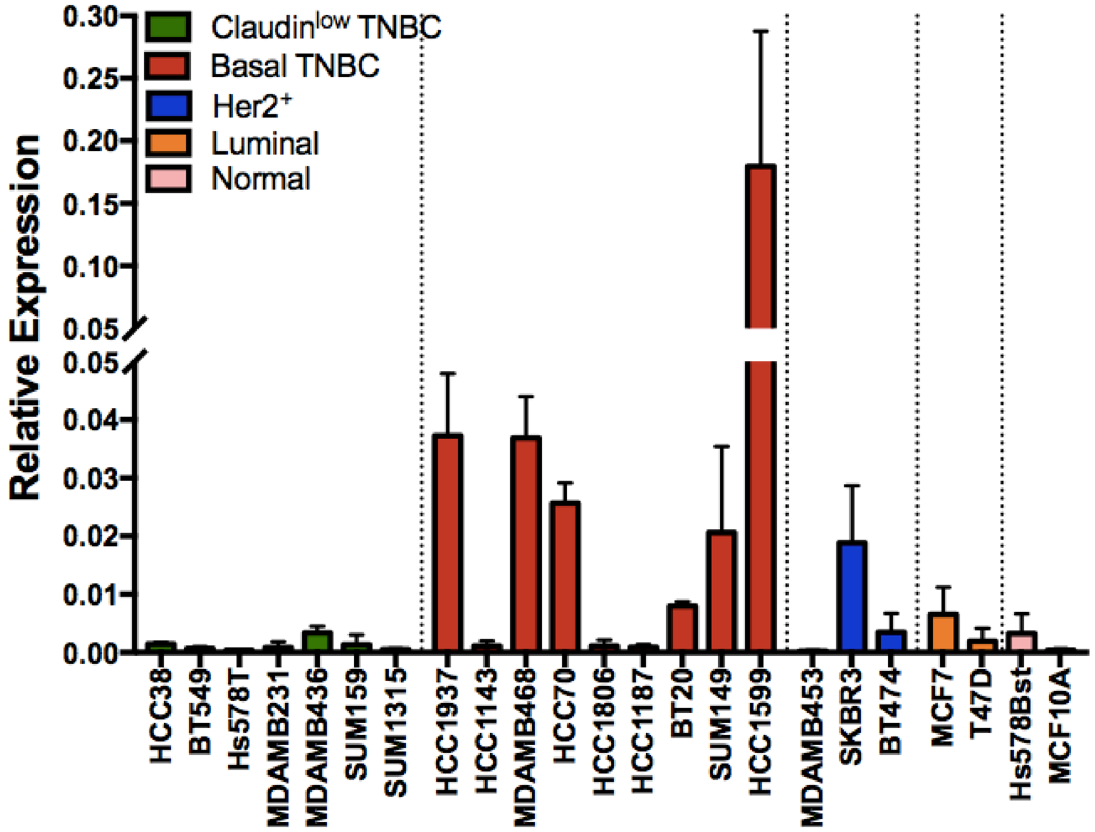

Supplemental Figure 4. NRAD1 is predominately expressed in basal-like breast cancer cell lines. NRAD1 expression in 21 cancerous, and two normal-like breast cell lines was determined by QPCR. PUM1 and ARF1 are used as reference genes in the panel due to target stability values across all 23 cell lines (n=4). Error bars represent standard deviation.

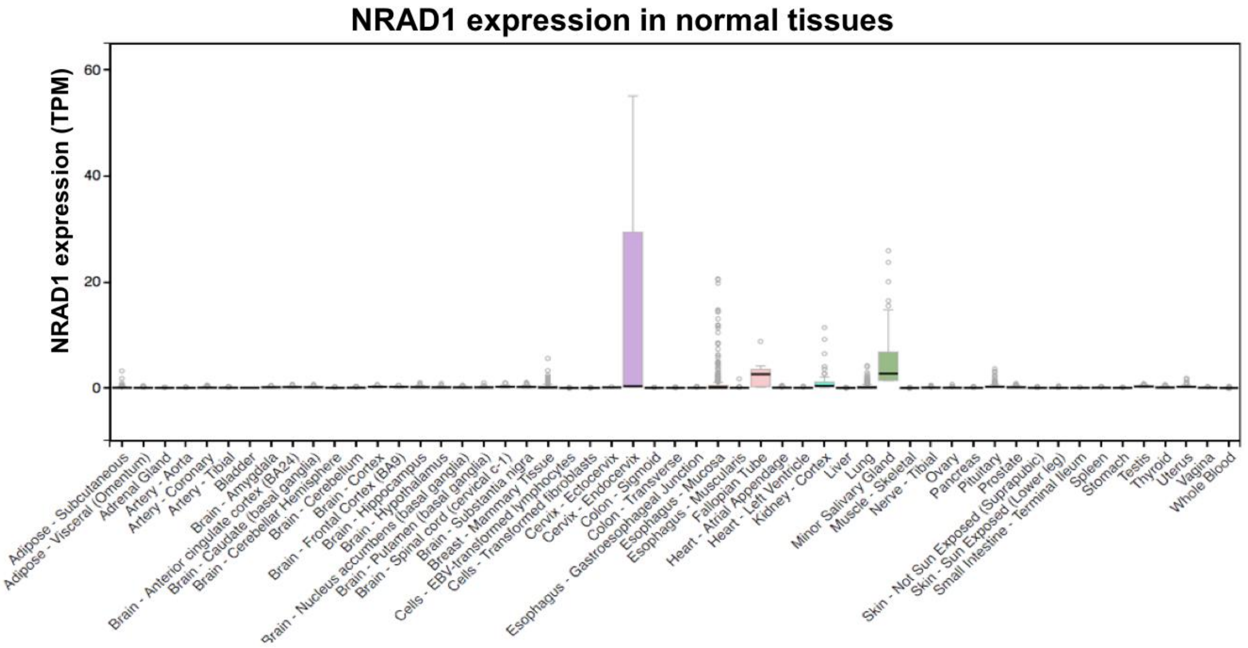

Supplemental Figure 5. NRAD1 is poorly expressed in most normal human tissues. The GTEx Portal (gtexportal.org) was used to assess NRAD1 expression levels across human tissues. The Genotype-Tissue Expression (GTEx) project is a resource database and associated tissue bank used to study the relationship between genetic variation and gene expression, and other molecular phenotypes, in multiple reference tissues.

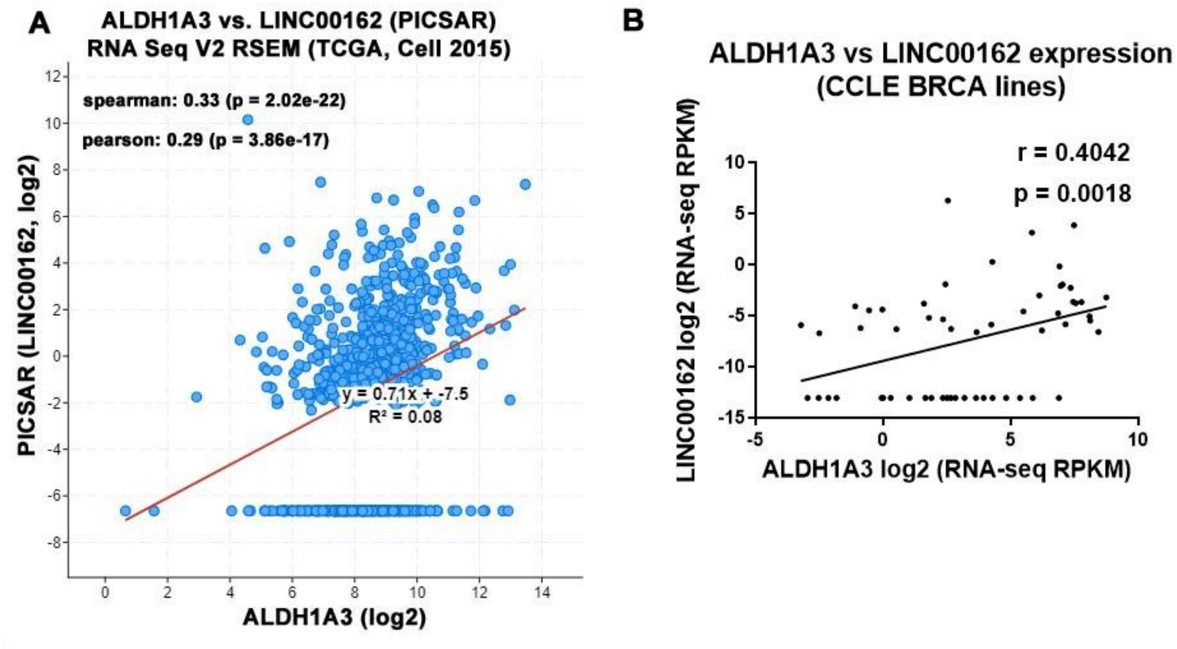

Supplemental Figure 6. LINC00162 expression correlation with ALDH1A3 expression in breast cancer patient tumors and cell lines. (A) RNA-seq co-expression of LINC00162 (PICSAR) and ALDH1A3 in the TCGA Cell 2015 dataset was retrieved with cBioportal. **(B)** RNA-seq co-expression of LINC00162 and ALDH1A3 in the Cancer Cell Line Encyclopedia (only breast cancer cell lines) was retrieved using the CCLE portal (r = pearson correlation).

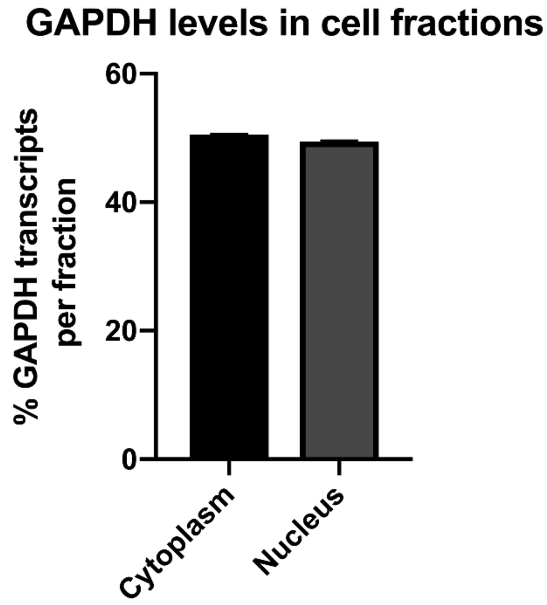

Supplemental Figure 7. GAPDH expression in cytoplasmic and nuclear compartments is nearly equal. Post cellular fractionization, RNA was isolated and cDNA synthesized. The GAPDH levels in the cytoplasmic and nuclear compartments were measured using QPCR in MDA-MB-468 cells. Transcript levels in each compartment is represented relative to the total levels of GAPDH in the cell, which is set to 100% for each n (i.e. levels of GAPDH are nearly equal in both compartments, n =3). Error bars represent standard deviation.

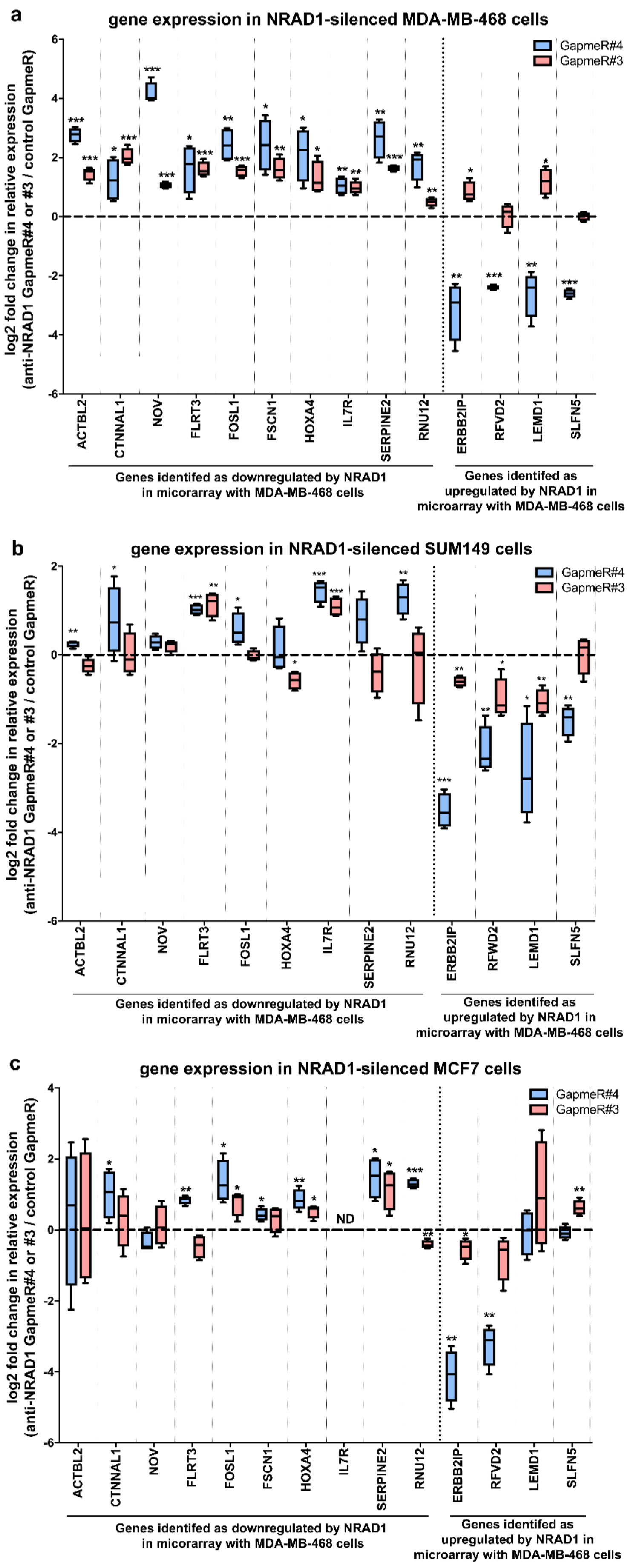
a
gene expression in NRAD1-silenced MDA-MB-468 cells
log2 fold change in relative expression
(anti-NRAD1 GapmeR#4 or #3 / control GapmeR)
GapmeR#4
GapmeR#3
ACTBL2
CTNNAL1
NOV
FLRT3
FOSL1
FSCN1
HOXA4
IL7R
SERPINE2
RNU12
ERBB2IP
RFVD2
LEMD1
SLFN5
Genes identifed as downregulated by NRAD1
in micorarray with MDA-MB-468 cells
Genes identifed as
upregulated by NRAD1 in
microarray with MDA-MB-468 cells
b
gene expression in NRAD1-silenced SUM149 cells
log2 fold change in relative expression
(anti-NRAD1 GapmeR#4 or #3 / control GapmeR)
GapmeR#4
GapmeR#3
ACTBL2
CTNNAL1
NOV
FLRT3
FOSL1
HOXA4
IL7R
SERPINE2
RNU12
ERBB2IP
RFWD2
LEMD1
SLFN5
Genes identifed as downregulated by NRAD1
in micorarray with MDA-MB-468 cells
Genes identifed as
upregulated by NRAD1 in
microarray with MDA-MB-468 cells
c
gene expression in NRAD1-silenced MCF7 cells
log2 fold change in relative expression
(anti-NRAD1 GapmeR#4 or #3 / control GapmeR)
GapmeR#4
GapmeR#3
ND
ACTBL2
CTNNAL1
NOV
FLRT3
FOSL1
FSCN1
HOXA4
IL7R
SERPINE2
RNU12
ERBB2IP
RFVD2
LEMD1
SLFN5
Genes identifed as downregulated by NRAD1
in micorarray with MDA-MB-468 cells
Genes identifed as
upregulated by NRAD1 in
microarray with MDA-MB-468 cells

Supplemental Figure 8. QPCR validation of a representative sampling of the microarray-identified NRAD1-regulated genes in MDA-MB-468, SUM149, and MCF7 cells. Log2 fold change of transcript levels in cells treated with anti-NRAD1-specific GapmeR#3 or #4 versus control GapmeR in MDA-MB-468 cells **(A),** SUM149 **(B),** and MCF7 cells **(C).** Expression is normalized to reference genes PUM1 and ARF1 and represented as fold change over GapmeR control-treated cells (n=4). Error bars represent standard deviation (ND = not detected, i.e. expression levels below quantification threshold).

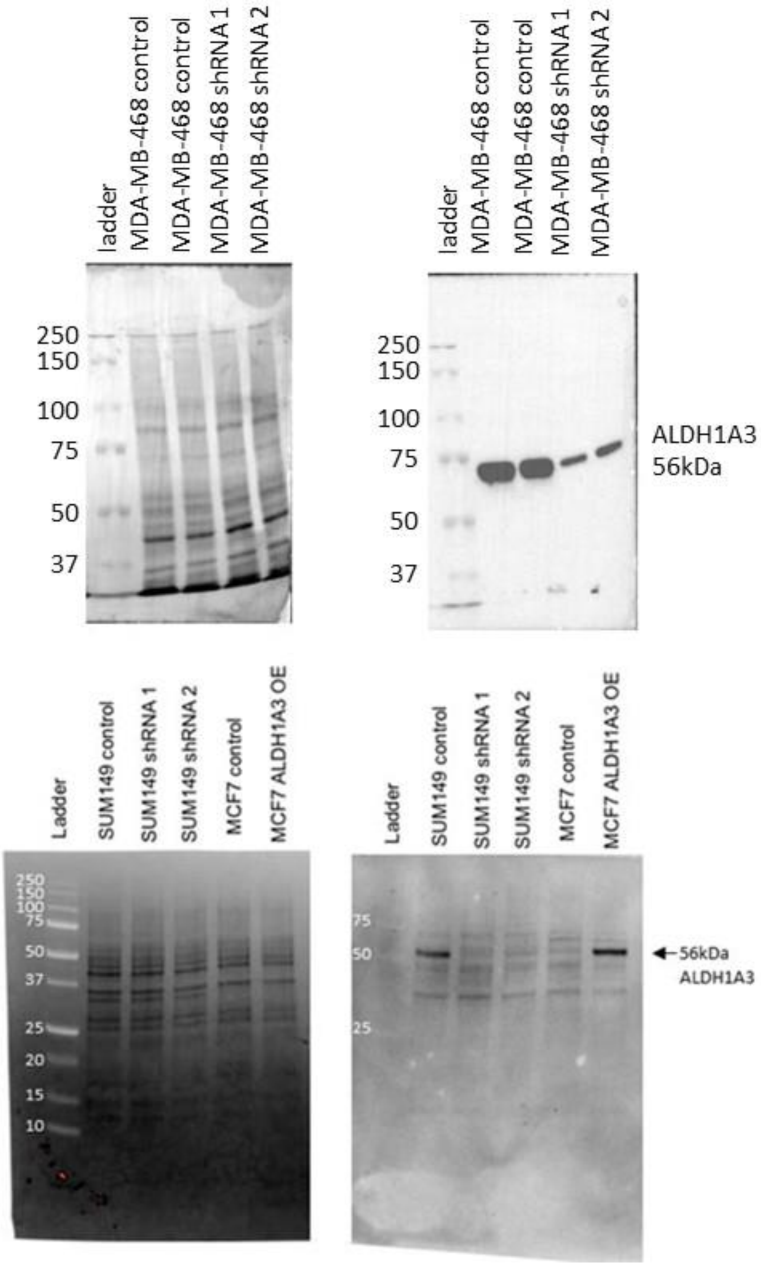

Supplemental Figure 9. Uncropped ALDH1A3 western blots (right) and total protein loading control blots (left).

Supplemental Tables

Supplemental Table 1. Primers used in QPCR, western antibody details, and GapmeR sequences.

Primer sequences	Forward	Reverse
PUM1	GGCGTTAGCATGGTGGAGTA	CATCCCTTGGGCCAAATCCT
ARF1	GTGTTCGCCAACAAGCAGG	CAGTTCCTGTGGCGTAGTGA
GAPDH	GGAGTCAACGGATTTGGTCGTA	TTCTCCATGGTGGTGAAGAC
B2M	AGGCTATCCAGCGTACTCCA	CGGATGGATGAAACCCAGACA
NRAD1 (LINC00284)	CCAGGGGATAAAACCCGCT	TAAGCACCAAGTCACGCTGC
ALDH1A1	TGTTAGCTGATGCCGACTTG	TTCTTAGCCCGCTCAACACT
ALDH1A3	TCTCGACAAAGCCCTGAAGT	TATTCGGCCAAAGCGTATTC
ZFAS1	CAACTACTAGAGCGCCTCGG	CCAAAGATGGCTTTCGCACC
SNHG6	CTGTCTTCCGATGTCGCTCT	CGGCATGACTAACGGCTCTT
LRCC75A-AS1	TTCCCGTTGTTATGGAGGGC	TCCAGTTCTCTCGGGTTTGC
AC004542.2	CAGAAAGGCCAGCCATACCA	ACCCCTGAGTACCCAGAGTAA
AC0093001	TCGCCAAGCAATTACCTACCA	CGGCTGCGGGTATATTCCAA
UCA1	CCGAGAGCCGATCAGACAAA	GGGATGGCCATTTGGAAGGA
TUG1	AGCGTGGGTGTACGTAAAGG	CCAAGGATTGGGGAACTGCT
NORAD	CTTAAGGGGCCTGGAAGGTG	AGAATGAAGACCAACCGCCC
MALAT1	GCAAAACGTGTGGCTGTCTT	GTGGCAAAATGGCGGACTTT
LINC-ROR	GAATCAGAGTGCTGGGCAGT	TCAGCAGCTCATGCCCTAAC
HULC	ACTCTGAAGTAAAGGCCGGAA	TGCCAGGAAACTTCTTGCTTG
HOTAIR	GGGACAGAAGGAAAGCCCTC	GAGTCAGAGTTCCCCACTGC
H19	GAGAGCTTGTGGGAGCCAAG	CCTACTCCACACTCCTCACTG
CYTOR	TTCCAACCTCCGTCTGCATC	GGGGGCTGAGTCGTGATTTT
TUNAR	ACCGGCAGCGTTATTGTTTC	CTAATCCCGAGCTTTCCCCC
GAS1RR	AAGGGGGCAACTGTATACGC	GGAGGTGCTTGATCACTGGT
NEAT1	CCTCCCTTTAACTTATCCATTCAC	TCTCTTCCTCCACCATTACCA
DANCR	AGGAGTTCGTCTCTTACGTCT	TGAAATACCAGCAACAGGACA
PART1	CAGGGTACGCCAACTATAGGAC	TTCAGCTTTCCAGAGCCAGT
LINC00511	TTCCCACAGGAAACCCACAC	CATCACCTGTCTCCTTGCCA
LINC01198	TGCCGAATAGCTCTGACCTG	GTTGAAAAAGGTGCAGTGGCA
LINC01354	ATGCACACATTCGAGGGGAA	CTGTGGAGGACGCTTGAGAG
LINC02159	TCCCATCGGCTTTTGGCTTT	GACACCTGTCCTGCCTCTTAG
AC022509.2	AGTTGGAACTTGTGACCAGCA	GCTTTCCAGCCTCACTTTGG
LINC00518	ACCTAACCTGCGAATGCTGT	GCCTAAACATTTGCTGCCCC

LINC00162 (PICSAR)	ATTGCTTAGGTGGGGAGCAC	TCATGACTGAGCTTTCCGTCG
RP11-120J1.1	TTCCCAAACCCCATGACTCTG	AGCCCATCACAGTGTTCCTT
LINC01315	CCAATTCCCCAGCGTTTTCC	GGCATCCACTTCATCGCTCA
AP000851.1	TGCTTGGCACATAGCATCCA	GGGTCCATGTTTTCCAAGACG
LNC-DPMI-1:1-2	CCCTGCATGTGCTAAGTGCT	GTGGAAAAATCCTGACATGGTGA
LINC01833	CTTTGTTGGGTTTGGGAGGC	AGGTGAGCTGGCGAATACTG
LINC00839	GGCCCAGATTGTTCCAGGAT	TGGTGAAAAGGCAGATCCCA
LINC00880	CGGGAAAGGTGTACCTCGTG	CCAGGGGCTTTGATCAACCT
LINP1	GACCAGGGCACTCTGTAAGG	GCAGTGGAGTCTGAAGTCCC
AP001626.1	CCCCAGTGGAGGAACCTTCT	GTATGGCAGCCAGGCGATT
AC016995.3	ACTCTGTCCTCTGTCACTGC	CTACTCACGTCCCCTTGGTC
LINC01956	AGTGTTGACTTGGGGACTGC	CAAAAGTCGCGCACTACCTG
LINC00707	CCCAGACATGACCCGATGAC	ATTTTGGTTTGCTGGCCCTG
LNC-NKX-1-2-1:1	GACCCTTCTGGTTTCCACAGA	GGATTTGGTTGGAAAGCCACT
LINC02188	TTGAAGGACCCCTGAATGGC	TCCACGGCTTTGTCTGTCTG
AC091053.1	GAAGCCAGCAAACATCTGAGAC	GAGAGGCCTTCCGCAAATCT
FOXP4-AS1	CTCTGTTTCCGTGGCAACCT	GACCTGGAGCTGTCATCGAG
DGCR5	CCATGGTTCGACGCCATTC	CCAGGGGGCCTTCTTTTTCT
SOX9-AS1	CAACATCTGCATTGGCGGAG	TGCTGAGGCTTCACTCATGG
LINC01819	CTAGGCACACTTGCCCTACC	GTGGGTCTACCCTGTATGCC
LINC00092	CCCATTCTCTTAGGCCCGTC	AGAAACATGCTTTGCGCTGG
LNC-DSC2-1:1	AGCTGCTCCTTATTTCTCCTGTAG	TTGCATGGAGAATGCGATGC
AC027031.2	GTGCAGGATCCGAAACAGGA	AACATGGCAAGCTGGATGGA
VIM-AS1	GCCCAGGCATTGAGTACCAT	CGACGTGTTGTCCTGATGGA
LINC02487	GTCTGTGTCCCTCAGAAGGC	AGAACCAGCGTTTCGGATGT
AC025154.2	AACCACAGGTCGCCACATAG	CAGCTGCCCTGTGTGAGTAA
AC009041.2	GTCCAACCGCGGGTCC	GGTTGAAAGGATCCGAGCCA
AC015712.4	AAGAACCAAGGTGCAACAGGA	GCACTCTTGAGGTTCCCCTG
CYP4Z1	GAATCCTGGGTTGGTCGAGG	AGGTTTCACAATCTGGCGGT
CTNNAL1	CCATGATGGCTCTCTTAGTCCA	ACCCATCCGTTATTTTCCATCTGA
EIF5A2	AGAACGGCTTCGTGGTACTG	CGTGCTTTCCCGTCTTGGA
FOSL1	CTGGTGCCAAGCATCAACAC	ACTGAGGGTAGGTCAGAGGC
FSCN1	GCAAGAATGCCAGCTGCTAC	ACAAACTTGCCATTGGACGC
IL7R	TTCTCTGTCGCTCTGTTGGTC	ACTGGGCCATACGATAGGCT
SERPINE2	ATTGAACTGCCCTACCACGG	GTGTGGGATGATGGCAGACA

SLITRK6	TGCTGCAGGGATAGTGGTTC	TGCACAGGACTGTTGTCTCTC
**Western blot antibodies**	**Details, catalogue number**	**Concentrations**
ALDH1A3	Mouse monoclonal antibody, clone OTI4E8. Origene catalogue number: TA502841	WB: 1/1000
**GapmeR Sequences**		
Control GapmeR (negative control A)	5'-AACACGTCTATACGC-3'	
NRAD1 GapmeR#3	5'-GCTGAACGCTGCCTTT-3'	
NRAD1 GapmeR#4	5'-CTTTGCTGAACTGATG-3'	

Supplemental Table 2. Summary of survival analyses completed on lncRNAs from Supplemental Figures 1, 2, and 3.

LncRNA	KM Plotter	TCGA (Cell 2015, cBioportal)	TCGA (BRCA, TANRIC)
RP11-120J1.1 (ENSG00000225472.1)	no data	no data	p = 0.176 (trend, high expression better survival)
AC022509.2 (ENSG00000256234.1)	no data	no data	p = 0.401
LINC00162 (PICSAR)	HR = 0.9, p = 0.54	HR = 1.53, p = 0.444	p = 0.817
LINC00284 (NRAD1)	HR = 1.66, p = 0.0025	HR = 2.36, p = 0.157	p = 0.404
LINC00511	no data	no data	p = 0.92
LINC00518	HR = 1.22, p = 0.22 (probe 1)<br>HR = 1.05, p = 0.79 (probe 2)	HR = 2.19, p = 0.172	p = 0.737
LINC01198 (ENSG00000231817.3)	HR = 0.57, p = 0.00069	no data	p = 0.925
LINC01354 (ENSG00000231768.1)	HR = 0.87, p = 0.41	no data	p =0.786
LINC02159 (ENSG00000253417.1)	no data	HR = 1.178, p = 0.762	p = 0.117 (trend, high expression better survival)
PART1	HR = 1.49, p = 0.0021 (probe 1)<br>HR = 0.77, p = 0.043 (probe 2)	HR =2.78, p = 0.071	p = 0.532

NRAD1 coding potential

	Metric	Raw result	Interpretation
	PRIDE reprocessing 2.0	0	non-coding ?
	Lee translation initiation sites	0	non-coding ?
	PhyloCSF score	-78.2965	non-coding ?
	CPAT coding probability	0.83%	non-coding ?
	Bazzini small ORFs	0	non-coding ?

Supplemental Table 3. NRAD1 is non-coding based on five metrics of protein-coding potential. Using online software lncipedia.org, the coding potential of NRAD1 was assessed by five metrics. The PRIDE reprocessing score analyzes the predicted open reading frames of a sequence against over 100 human proteomics mass spectra; score of 0 indicates no hits. The Lee translation initiation sites are mapped using lactimidomycin, an initiating ribosome inhibitor (compared to a no treatment control); a score of 0 means no difference (i.e. no translation). The PhyloCSF algorithm generates probabilistic models of coding potential based on Codon Substitution Frequencies; a score under +60.00 is likely to be non-coding (lower number increases odds that the sequence is non-coding). CPAT assesses ORF lengths (a long non-putative ORF is unlikely to be observed by random chance in a non-coding sequence) and ORF coverage (the ratio of ORFs to transcript lengths). Bazzini Small ORFs tests if small ORFs in the sequence are translated through ribosomal profiling. A score of 0 indicates no translation detected.
